# *Bifidobacterium Breve Yang08* Alleviates Atopic Dermatitis By Enriching Akkermansia Muciniphila and Inhibiting Neutrophil Extracellular Traps Formation In Mice

**DOI:** 10.1002/advs.202518588

**Published:** 2026-02-08

**Authors:** Yanqiang Shi, Shuang He, Chengyi Li, Hung Chan, Zhenfeng Liu, Bin Yang, Qing Li

**Affiliations:** ^1^ Dermatology Hospital Southern Medical University Guangzhou China; ^2^ The First School of Clinical Medicine, Southern Medical University Guangzhou China

**Keywords:** Akkermansia muciniphila, atopic dermatitis, Bifidobacterium breve, neutrophil extracellular traps, neutrophil

## Abstract

Atopic dermatitis (AD) is linked to gut microbiota dysbiosis, yet the mechanisms connecting specific commensals to cutaneous immunoregulation remain elusive. We observed reduced *Bifidobacterium breve* (*B. breve*) abundance in AD patients. A new *B. breve* strain was isolated from human stools and nomenclated as *Yang08*. In MC903‐induced AD‐like mouse models, *Yang08* outperformed a standard strain, ameliorating disease severity, including reduced ear thickening, epidermal hyperplasia, and mast cell infiltration in a manner dependent on viable bacteria and an intact gut microbiota. Antibiotic‐mediated microbiota depletion abrogated its efficacy, while fecal microbiota transfer from *Yang08*‐treated mice conferred protection, confirming microbial remodeling as essential. Metagenomics revealed *Yang08* specifically enriched *Akkermansia muciniphila*, which was required for therapeutic effects in germ‐free mice. Mechanistically, *Yang08* abolished both neutrophil influx and NET deposition in lesions, with ex vivo experiments showing blunted NETosis capacity. Its therapeutic benefits were reversed by neutrophil depletion, NET degradation, or PAD4 inhibition. Overall, *Yang08* alleviates AD by enriching *A. muciniphila* and inhibiting skin NETosis, emerging as a promising prophylactic candidate prevention for AD prevention.

## Graphical abstract

A novel *Bifidobacterium breve* strain, *Yang08*, is isolated to counter its depletion in atopic dermatitis. In mice, live *Yang08* requires an intact gut microbiota to exert protection, specifically enriching *Akkermansia muciniphila* and potently inhibiting pathogenic neutrophil activation and NETosis in the skin. These positions *Yang08* as a promising live biotherapeutic for AD.

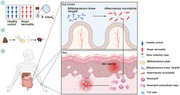

**Abbreviations**
A. muciniphila: Akkermansia muciniphila
ABX: antibiotics cocktail
AD: atopic dermatitis
B. breve: Bifidobacterium. breve
CitH3: citrullinated histone H3
E. coli: Escherichia coli MG1655
ELISA: Enzyme‐linked immunosorbent assay
GSEA: Gene Set Enrichment Analysis
LEfSe: Linear Discriminant Analysis Effect Size
MC903: calcipotriol
MPO: Myeloperoxidase
NET: neutrophil extracellular trap
PCA: Principal component analysis

## 1 Introduction

Atopic dermatitis (AD) is a chronic inflammatory skin disorder characterized by recurrent eczematous lesions and intense pruritus. Although it commonly manifests in infancy, its prevalence spans both pediatric and adult populations [1, 2]. The pathogenesis of AD is now understood to be multifactorial, involving a complex interplay of genetic predisposition, impaired epidermal barrier function, microbial dysbiosis, immune dysregulation, and neuroimmune activation [3, 4, 5]. In recent years, therapeutic strategies have evolved and advanced beyond conventional immunosuppressants. The advent of topical phosphodiesterase‐4 (PDE‐4) inhibitors and Janus kinase (JAK) inhibitors has broadened the treatment armamentarium for mild‐to‐moderate disease [6]. For moderate‐to‐severe AD, clinical management has been revolutionized by targeted biologics, such as anti‐IL‐4/IL‐13 therapies, and small molecule inhibitors targeting JAK/STAT signaling cascade [7]. Nevertheless, significant limitations persist, including partial or transient responses, disease relapse upon treatment cessation, and long‐term safety concerns, particularly with JAK inhibitors [8]. These challenges highlight the urgent need to investigate novel therapeutic targets and mechanisms.

Emerging evidence implicates the gut microbiota in the pathogenesis of AD via the gut‐skin axis, wherein intestinal microbes modulate cutaneous immunity and inflammation [9]. Consequently, therapeutic strategies like fecal microbiota transplantation (FMT) and probiotic supplementation have garnered substantial interest for their potential in managing AD [10, 11, 12]. Patients with AD consistently exhibit gut microbiota dysbiosis, notably a reduced abundance of *Bifidobacterium* [13]. However, the clinical translation of microbiome‐targeted interventions is hampered by an incomplete understanding of strain‐specific mechanisms and inconsistent therapeutic outcomes [10]. Specifically, most microbiome research in AD has focused on suppressing Th2 differentiation or enhancing regulatory T cells (Tregs) [9]. However, the role of the gut microbiota in modulating innate effector mechanisms—particularly the recruitment and activation of neutrophils—remains largely unexplored in the context of AD.

Neutrophils are increasingly recognized as key drivers of AD pathology. Neutrophils release neutrophil extracellular traps (NETs)—web‐like structures of decondensed chromatin and granule proteins—which amplify inflammation [14, 15, 16]. Recent studies suggest that NETs can potentiate Th2‐driven inflammation and compromise skin barrier integrity in AD [17, 18, 19]. Despite this, it remains unknown whether commensal gut bacteria can regulate cutaneous NETosis and whether this pathway can be harnessed therapeutically.

Building on our previous work demonstrating that bacteria depleted in disease states can serve as prophylactic agents [20, 21, 22, 23, 24], we identified that *Bifidobacterium breve* (*B. breve*) is significantly reduced in patients with AD compared to healthy subjects. In this study, we address key translational barriers by identifying and mechanistically validating *B. breve Yang08*, a novel strain isolated from the gut of a healthy human. We demonstrate that administration of live *Yang08* effectively ameliorates dermatitis in a gut microbiota–dependent mechanism. Specifically, *Yang08* enriches the beneficial commensal *Akkermansia muciniphila* (*A. muciniphila*) while uniquely suppressing neutrophil chemotaxis and subsequent NETs formation. This action disrupts a previously underexplored inflammatory amplification loop that drives cutaneous inflammation. These findings establish a robust mechanistic framework for the therapeutic potential of *Yang08*, and provide a scientific foundation for its future clinical application to improve outcomes in AD.

## 2 Results

### 2.1 Breve is Depleted in Atopic Dermatitis Patients and the Yang08 Attenuates Murine Dermatitis

**FIGURE 1:**
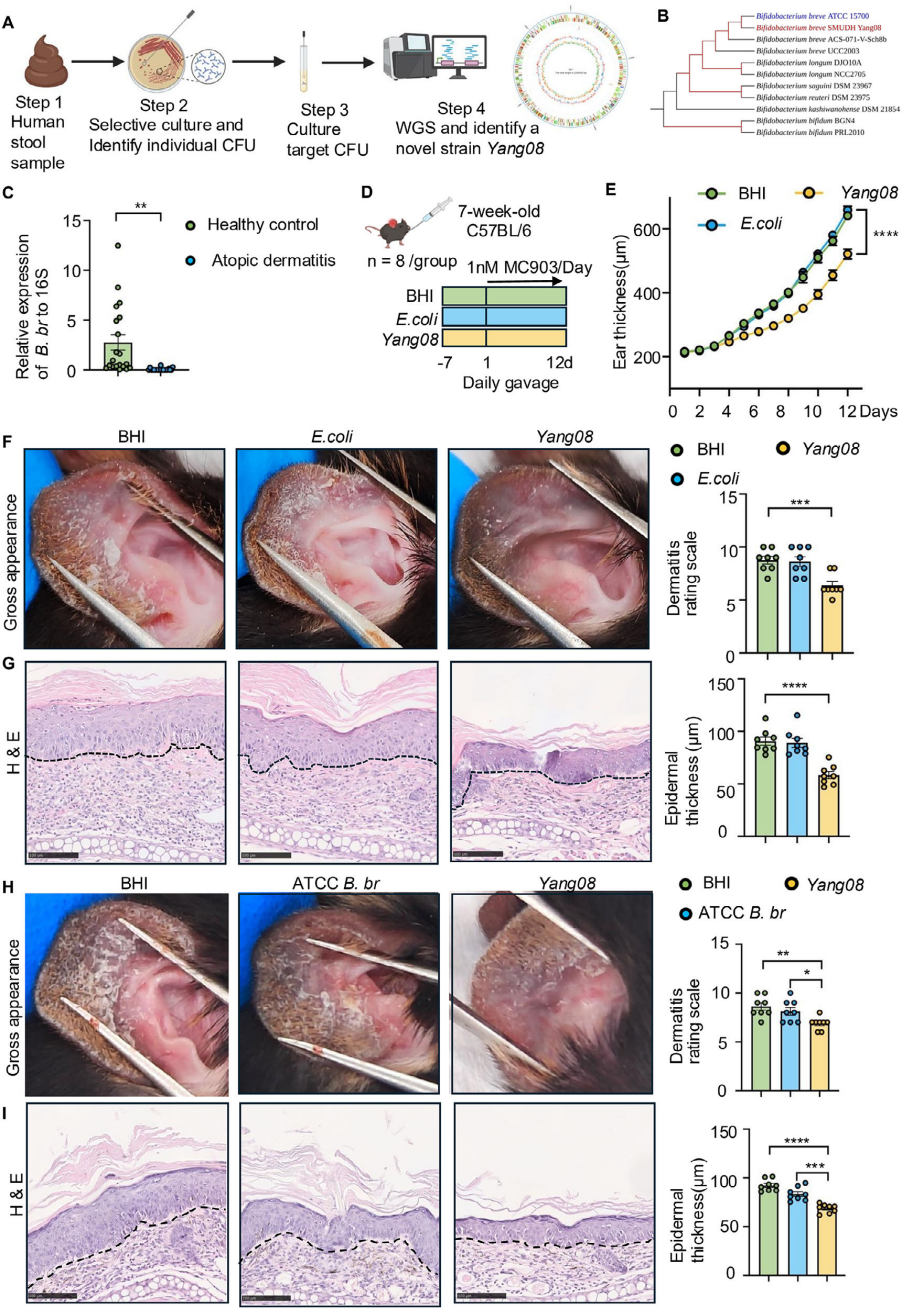
*B. breve* is depleted in atopic dermatitis (AD) patients and the *Yang08* attenuates murine dermatitis. (A) Schematic of the isolation and identification process for the novel *B. breve* strain *Yang08* from a healthy donor. (B) Phylogenetic tree based on core genomes, showing the position of *Yang08* (highlighted in red) relative to other *B. breve* reference strains. Red branches indicate bootstrap support of 100. (C) Quantification of fecal *B. breve* abundance via RT‐qPCR in healthy controls (HC) and atopic dermatitis (AD) patients (*n* = 20 per group). ^**^ *P *< 0.01 for AD vs. HC group by unpaired Student's t‐test. (D) Experimental timeline of the MC903‐induced AD model with indicated treatments. (*n* = 8 mice per group) (E) Ear thickness measurement over the course of MC903 challenge. ^****^ *P *< 0.0001 for *Yang08* vs. BHI group by two‐way ANOVA. (F) Representative macroscopic images of ears and clinical dermatitis scores on day 12. (G) Representative H&E‐stained sections of ear skin and quantification of epidermal thickness. Scale bar, 100 µm. For (F, G), ^***^ *P *< 0.001, ^****^ *P *< 0.0001 for *Yang08* vs. BHI group by one‐way ANOVA with Dunnett's test. (H, I) Comparison of therapeutic efficacy between *B. breve Yang08* and the standard ATCC 15700 strain. (*n* = 8 mice per group) (H)Representative macroscopic images of ears and clinical dermatitis scores on day 12. (I) Representative H&E‐stained sections of ear skin and quantification of epidermal thickness. Scale bar, 100 µm. For (H‐I), ^*^ *P *< 0.05, ^***^ *P *< 0.001, ^****^ *P *< 0.0001 for *Yang08* vs. BHI or ATCC *B. breve* group by one‐way ANOVA with Dunnett's test. All data are presented as mean ± SEM.

It has been reported that *Bifidobacterium* is depleted in patients with AD [13], then we isolated a novel *B. breve* strain, designated SMUDH*Yang08* (*Yang08*), from healthy donor specimens using *Bifidobacterium* selective agar under anaerobic conditions (Figure 1A and SFigure 1A). Whole‐genome sequencing and phylogenetic analysis confirmed *Yang08* as a novel bacterial strain (Figure 1B). To evaluate the abundance of *B. breve* in AD patients, we collected the fecal samples from 20 patients with AD and from age‐ and gender‐matched healthy controls. DNA extraction followed by RT‐qPCR revealed a significant reduction in fecal *B. breve* abundance in AD patients as compared with healthy individuals (*P < *0.01; Figure 1C).

To evaluate the therapeutic potential of *Yang08*, we employed an MC903‐induced murine AD‐like model. Mice were pretreated for seven days via daily oral gavage with sterile BHI broth (vehicle control), *E. coli* (negative control; 2 × 10^8^ CFU), or *Yang08* (2 × 10^8^ CFU) (Figure 1D). *Yang08*‐treated mice exhibited significantly attenuated ear thickness relative to the BHI groups (*P < *0.0001; Figure 1E), along with improvements in gross skin appearance and dermatitis scores (*P < *0.001; Figure 1F). Histological analysis revealed reduced epidermal thickness on H&E staining (*P < *0.0001; Figure 1G) and diminished mast cell infiltration, as shown by toluidine blue staining (*P < *0.0001; SFigure 1B and C).

We further compared the therapeutic efficacy of strain *Yang08* with that of the standard *B. breve* ATCC strain in vivo (SFigure 1D). Although *Yang08* did not show a statistically significant difference in ear thickness change compared to the ATCC strain (SFigure 1E), it demonstrated significant improvements in dermatitis scores (*P < *0.05; Figure 1H), epidermal thickness (*P < *0.001; Figure 1I), and mast cell infiltration (*P < *0.05; SFigure 1F). Collectively, these findings demonstrate the superior therapeutic efficacy of the *Yang08* strain, confirming that *B. breve* is depleted in patients with AD and that supplementation with *Yang08* attenuates murine dermatitis.

### 2.2 *Yang08* Mediated Protection Against Dermatitis Requires Live Bacteria

**FIGURE 2:**
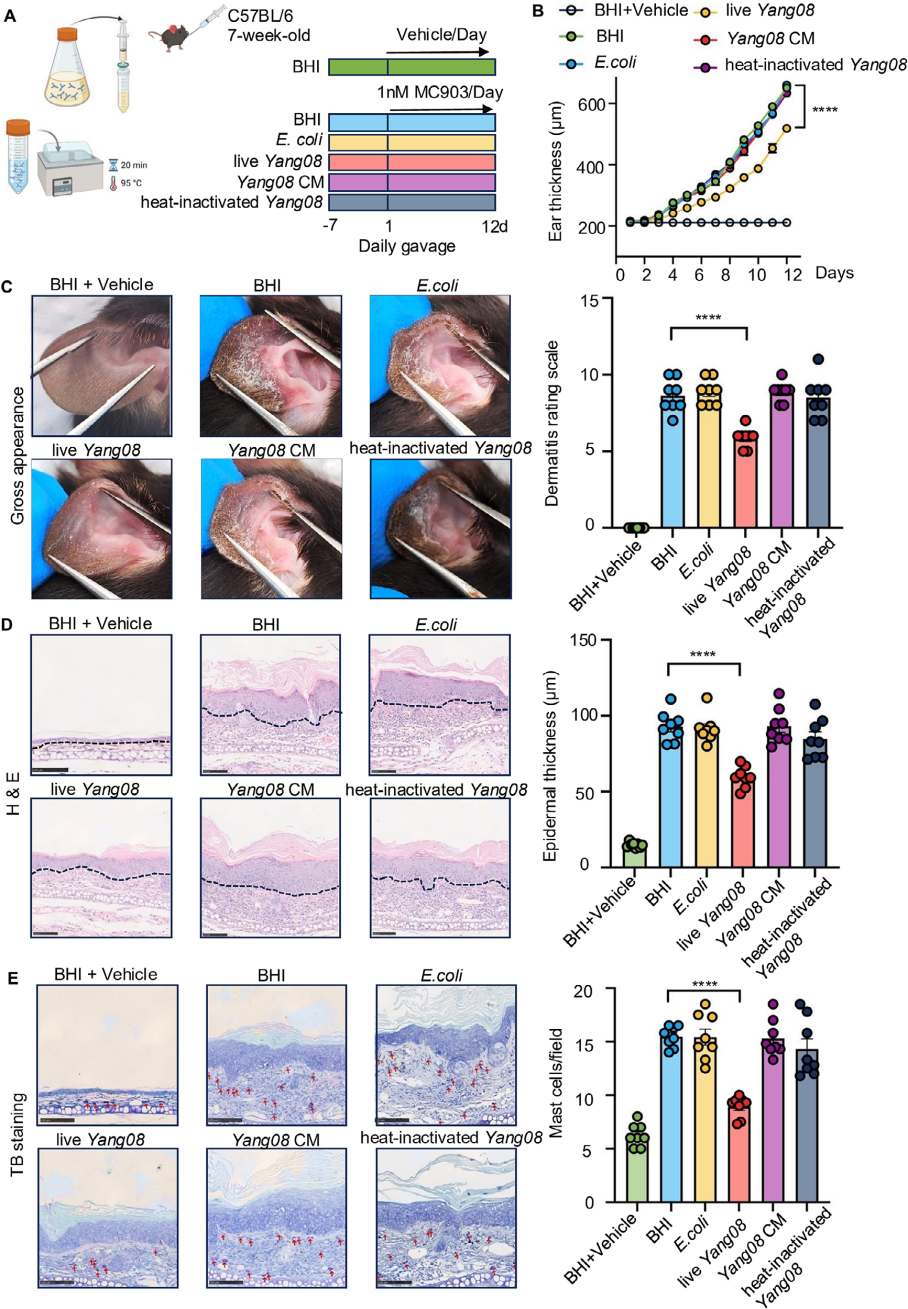
*Yang08* alleviates dermatitis depending on live bacteria (A) Schematic of the experimental design to test the activity of different *Yang08* components. (B–E) Mice were treated as indicated prior to and during MC903 challenge (*n* = 8 mice per group). (B) Ear thickness over time in mice treated with different *Yang08* components. ^****^ *P *< 0.0001 for live *Yang08* vs. BHI group by two‐way ANOVA. (C) Representative macroscopic images of ears and clinical dermatitis scores on day 12. (D) Representative H&E‐stained sections of ear skin and quantification of epidermal thickness. Scale bar, 100 µm. (E) Representative toluidine blue‐stained sections of ear skin and quantification of mast cells per HPF. Red arrows indicate mast cells. Scale bar, 100 µm. For (C–E), ^****^ *P *< 0.0001 for live *Yang08* vs. BHI group by one‐way ANOVA with Dunnett's test. All data are presented as mean ± SEM.

To elucidate the active component mediating *Yang08*'s therapeutic effect, mice were assigned to six treatment groups: BHI with vehicle, BHI with MC903, live *E. coli* with MC903, live *Yang08* with MC903, *Yang08* culture medium with MC903, and heat‐inactivated *Yang08* with MC903 (Figure 2A). Compared with the BHI group, administration of live *Yang08* resulted in a significant attenuation of ear thickness, whereas no significant differences were observed with the culture medium of *Yang08* or heat‐inactivated *Yang08* (*P < *0.0001; Figure 2B). This viability‐dependent benefit extended to dermatitis severity scores (*P < *0.0001; Figure 2C). Histopathological analyses corroborated these findings: only live *Yang08* mitigated epidermal hyperplasia (*P < *0.0001; Figure 2D) and suppressed mast cell infiltration (*P < *0.0001; Figure 2E). Collectively, these results demonstrate that *Yang08*’s protective effects are contingent upon bacterial viability and cannot be recapitulated by *Yang08's* culture medium or heat‐inactivated *Yang08*.

### 2.3 *Yang08*‐Mediated Amelioration of Dermatitis Requires an Intact Gut Microbiota

**FIGURE 3:**
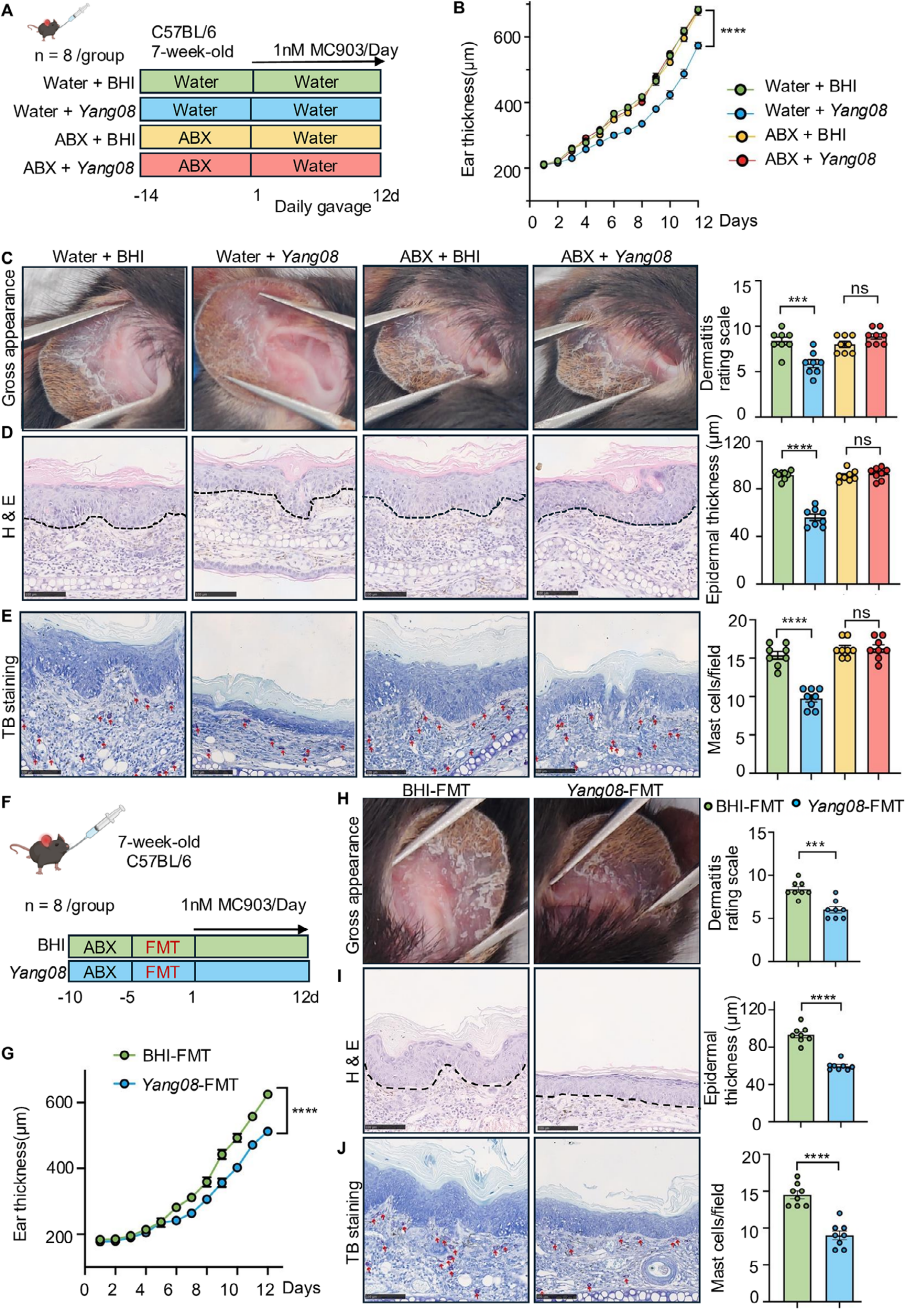
*Yang08*‐mediated amelioration of dermatitis requires an intact gut microbiota. (A) Schematic of the antibiotic (ABX) depletion experiment. (B–E) Effects of *Yang08* in mice with or without ABX‐induced microbiota depletion (*n* = 8 mice per group). (B) Ear thickness over time in mice. ^****^ *P *< 0.0001 for Water + *Yang08* vs. Water + BHI by two‐way ANOVA. (C) Representative macroscopic images of ears and clinical dermatitis scores on day 12. (D) Representative H&E‐stained sections of ear skin and quantification of epidermal thickness. Scale bar, 100 µm. (E) Representative toluidine blue‐stained sections of ear skin and quantification of mast cells per HPF. Red arrows indicate mast cells. Scale bar, 100 µm. For (C–E), ^***^ *P *< 0.001 for Water + *Yang08* vs. Water + BHI; ns, not significant for ABX + *Yang08* vs. ABX + BHI by unpaired t‐test. (F) Schematic of the fecal microbiota transplantation (FMT) experiment. (G–J) Therapeutic effects of FMT from *Yang08*‐treated donors (*Yang08*‐FMT) vs. BHI‐treated donors (BHI‐FMT) (*n* = 8 mice per group). (G) Ear thickness over time in mice treated with *Yang08*‐ or BHI‐ FMT. ^****^ *P *< 0.0001 for *Yang08*‐FMT vs. BHI‐FMT group by two‐way ANOVA. (H) Representative macroscopic images of ears and clinical dermatitis scores on day 12. (I) Representative H&E‐stained sections of ear skin and quantification of epidermal thickness. Scale bar, 100 µm. (J) Representative toluidine blue‐stained sections of ear skin and quantification of mast cells per HPF. Red arrows indicate mast cells. Scale bar, 100 µm. For (H–J), ^***^ *P *< 0.001, ^****^ *P *< 0.0001 for *Yang08*‐FMT vs. BHI‐FMT group by unpaired t‐test. All data are presented as mean ± SEM.

Given that only live *Yang08* conferred therapeutic benefits, we hypothesized that its anti‐inflammatory effects depend on interactions with the gut microbiota. To test this, mice were pretreated with ABX or vehicle in drinking water for two weeks to deplete the gut microbiota [25, 26]. Subsequently, mice were treated with BHI or *Yang08* (oral gavage) in the absence of antibiotics. The experiment comprised four groups: water + BHI, water + *Yang08*, ABX + BHI, or ABX + *Yang08* (Figure 3A). As expected, water + *Yang08* group showed significantly attenuated ear thickness compared to the water + BHI group (*P < *0.0001; Figure 3B). However, this protective effect was entirely abolished in ABX‐treated cohorts (*P *> 0.05; Figure 3B). Similarly, ABX pretreatment eliminated *Yang08*'s beneficial effects on dermatitis severity scores, epidermal thickness, and mast cell infiltration (all *P *> 0.05; Figure 3C–E).

To determine whether the *Yang08*‐reconfigured gut microbiota is sufficient to relieve dermatitis, we performed an FMT experiment (Figure 3F). Compared with recipients of BHI‐FMT, mice that received *Yang08*‐FMT exhibited a marked reduction in ear thickness (*P < *0.0001; Figure 3G), clinical severity score (*P < *0.001; Figure 3H), epidermal hyperplasia (*P < *0.0001; Figure 3I), and mast cell infiltration (*P < *0.0001; Figure 3J).

Collectively, these data demonstrate that *Yang08* alleviates dermatitis in a gut microbiota‐dependent manner, as its therapeutic effect is both abolished by microbiota depletion and transferred by transplantation of the modified microbiota, thereby establishing a causal role for microbial remodeling in mediating the observed protection.

### 2.4 *Yang08*Reshapes the Gut Microbiome and Enriches *A. Muciniphila*

**FIGURE 4:**
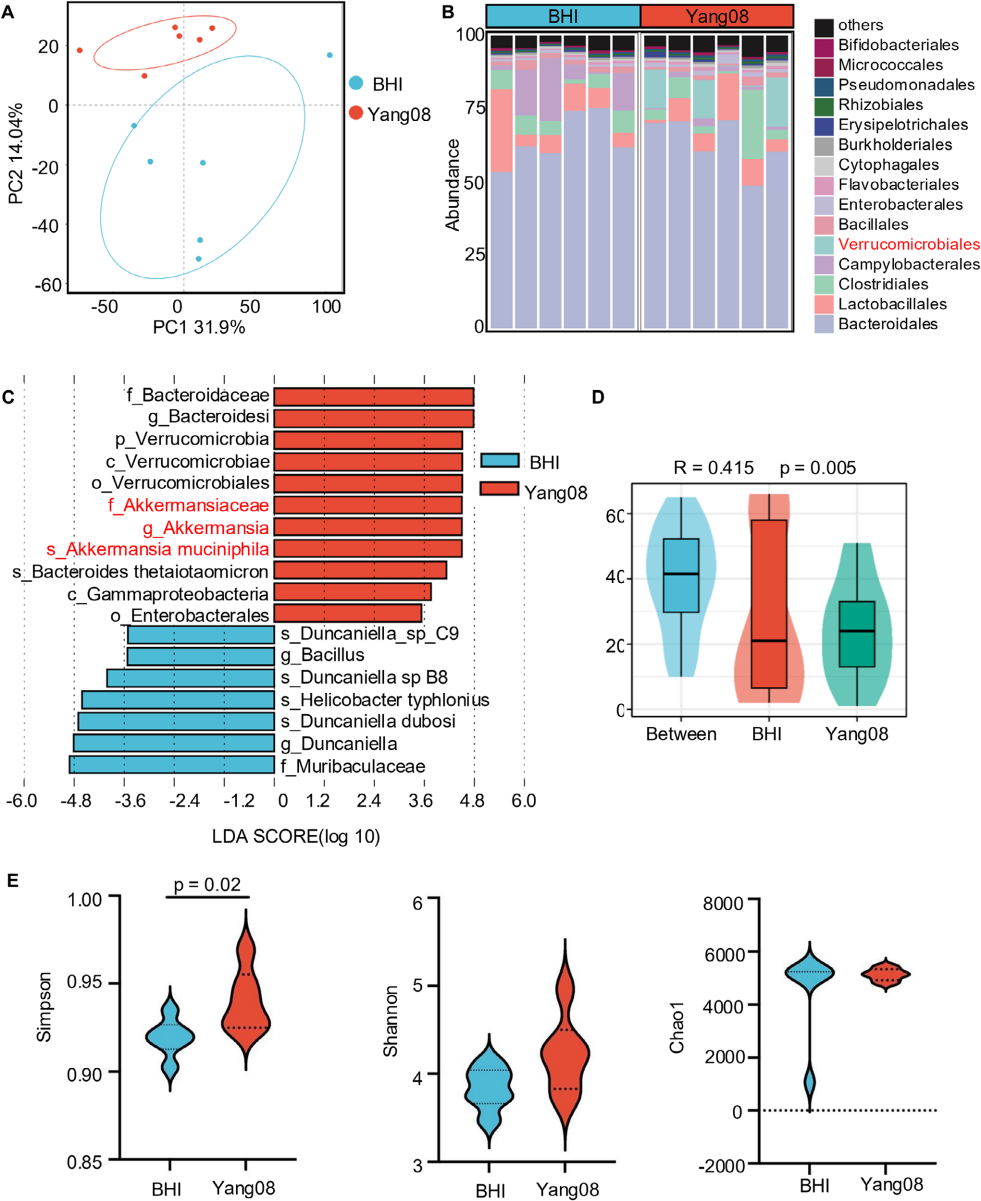
*Yang08* remodels the gut microbiome and enriches *A. muciniphila* (A) Principal component analysis (PCA) of shotgun metagenomic sequencing data from fecal samples (*n* = 6 mice per group). (B) Taxonomic composition profiling at the order level. (C) Linear discriminant analysis Effect Size (LEfSe) identifying *A. muciniphila* as the most discriminatively enriched taxon in *Yang08*‐treated mice (LDA score>4.5, *P*=0.0039). (D) Beta‐diversity analysis (ANOSIM, R=0.415, *P*=0.005). (E) Alpha‐diversity indices (Chao1, Shannon, Simpson) by unpaired t‐test (Simpson index). Data are presented as mean ± SEM.

To investigate the gut microbiota‐dependent efficacy of *Yang08*, we performed shotgun metagenomic sequencing on fecal samples, enabling high‐resolution taxonomic profiling at the species level. Principal component analysis (PCA) showed distinct clustering between *Yang08*‐treated and control mice, indicating marked restructuring of the microbial community (Figure 4A). Taxonomic profiling at the order level revealed compositional shifts between cohorts, including in the order *Verrucomicrobiales* (Figure 4B). Notably, Linear Discriminant Analysis Effect Size (LEfSe) identified that *A. muciniphila* as the most significantly enriched taxon in *Yang08*‐treated mice as compared with the control group (LDA = 4.50, *P* = 0.0039; Figure 4C), demonstrating that the signal was driven primarily by *A. muciniphila* rather than by other members of *Verrucomicrobiales*. Beta‐diversity analysis confirmed robust intergroup separation (ANOSIM R = 0.415, *P* = 0.005; Figure 4D). Alpha‐diversity analysis showed a significant increase in Simpson diversity (*P < *0.05), with no significant changes in Chao1 richness or Shannon index (Figure 4E). Collectively, these data indicate that *Yang08* remodels the gut microbiota, leading to a specific enrichment of *A. muciniphila*—a well‐characterized beneficial commensal associated with immune regulation.

### 2.5 *Muciniphila* is Required for *Yang08’*s Efficacy in ABX‐Treated and Germ‐Free Mice

**FIGURE 5:**
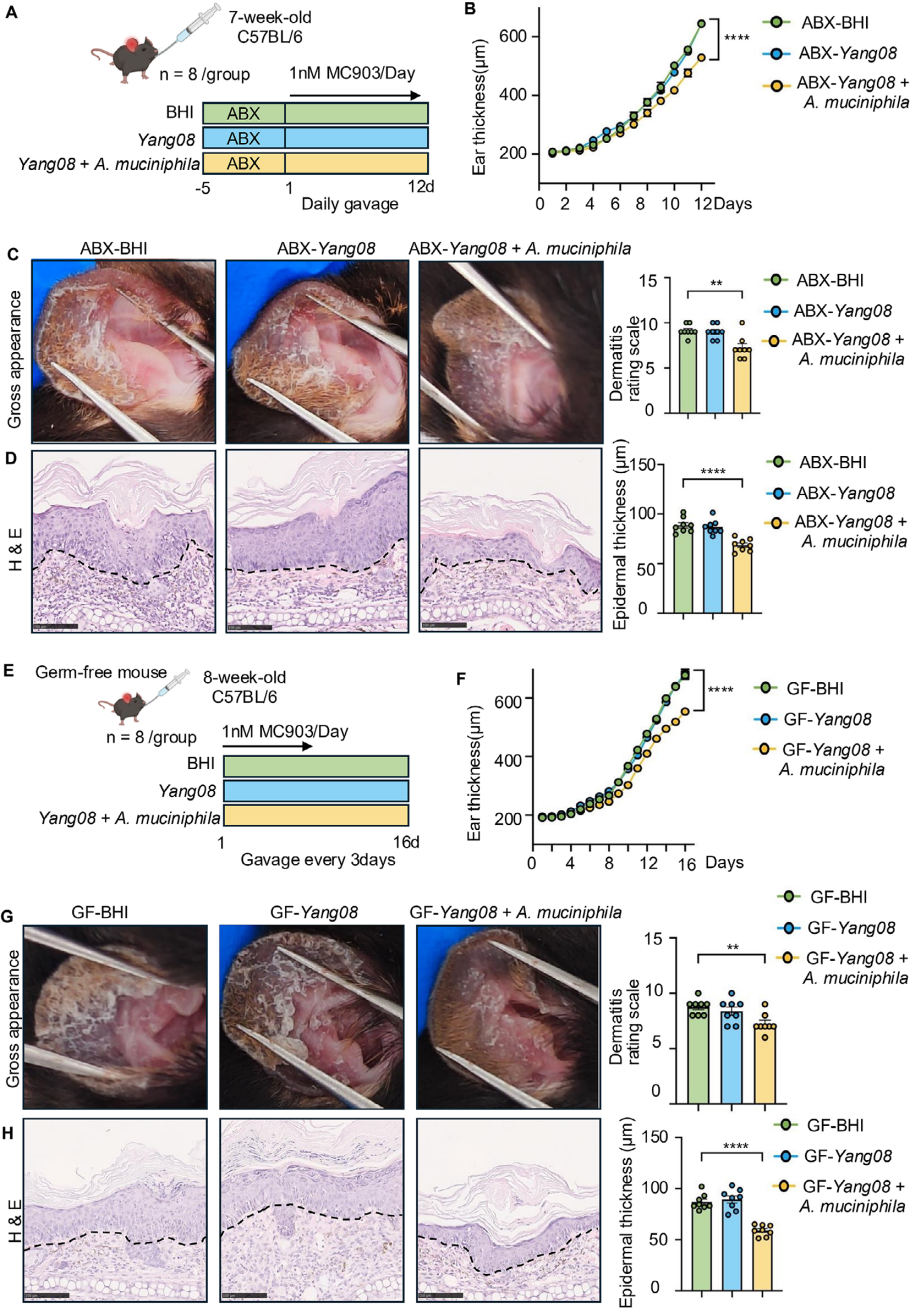
*A. muciniphila* is required for *Yang08*’s efficacy in ABX‐treated and germ‐free mice (A) Schematic of the experiment in antibiotic‐treated (ABX) mice. (B‐D) Disease parameters in ABX‐treated mice receiving BHI, *Yang08*, or *Yang08* + *A. muciniphila* (*n* = 8 mice per group). (B) Ear thickness over time in ABX‐treated mice. ^****^ *P *< 0.0001 for ABX‐ *Yang08* + *A. muciniphila* vs. ABX‐BHI by two‐way ANOVA. (C) Representative macroscopic images of ears and clinical dermatitis scores on day 12. (D) Representative H&E‐stained sections of ear skin and quantification of epidermal thickness. Scale bar, 100 µm. For (C‐D), ^**^ *P *< 0.01, ^****^ *P *< 0.0001 for ABX‐*Yang08* + *A. muciniphila* vs. ABX‐BHI group by one‐way ANOVA with Dunnett's test. (E) Schematic of the experiment in germ‐free (GF) mice. (F–H) Disease parameters in GF mice receiving BHI, *Yang08*, or *Yang08* + *A. muciniphila* (*n* = 8 mice per group). (F) Ear thickness over time in GF mice. ^****^ *P *< 0.0001 for *Yang08* + *A. muciniphila* vs. BHI group by two‐way ANOVA. (G) Representative macroscopic images of ears and clinical dermatitis scores on day 16. (H) Representative H&E‐stained sections of ear skin and quantification of epidermal thickness. Scale bar, 100 µm. For (G, H), ^**^ *P *< 0.01, ^****^ *P *< 0.0001 for *Yang08* + *A. muciniphila* vs. BHI group by one‐way ANOVA with Dunnett's test. All data are presented as mean ± SEM.

To directly test the hypothesis that *Yang08*’s therapeutic effect is mediated through its specific enrichment of A. muciniphila, we employed gnotobiotic models. In ABX‐treated mice, where the resident microbiota was ablated (Figure 5A), oral administration of *Yang08* alone failed to ameliorate dermatitis. In contrast, co‐administration of *Yang08* with *A. muciniphila* fully restored therapeutic efficacy, as evidenced by reductions in ear thickness (*P < *0.0001; Figure 5B), clinical scores (*P < *0.01; Figure 5C), epidermal hyperplasia (*P < *0.0001; Figure 5D), and mast cell infiltration (*P < *0.0001; SFigure 2A). We validated this essential synergy in genuine germ‐free mice (Figure 5E), where *Yang08* monotherapy again showed no therapeutic benefit, while the combination of *Yang08* and *A. muciniphila* successfully conferred protection across all measured disease parameters (*P < *0.01 for clinical scores, *P < *0.0001 for others; Figure 5F–H and SFigure 2B). Collectively, these results demonstrate that the presence of *A. muciniphila* is indispensable for *Yang08* to confer its full therapeutic effect against AD.

### 2.6 The Therapeutic Efficacy of *Yang08* Depends on Functional Neutrophils and the Suppression of NET Formation

To validate *Yang08*'s therapeutic benefits, we assessed systemic and local inflammatory responses. *Yang08* significantly reduced plasma total IgE levels (*P < *0.01; SFigure 3A) and suppressed spontaneous scratching behavior (*P < *0.01; SFigure 3B). Flow cytometry revealed markedly decreased infiltration of IL‐4‐producing CD4+ T cells in lesional skin (*P < *0.0001; SFigure 3C and D). Transcriptomic analysis further supported these findings, showing clear separation between groups by PCA, differential expression of 2,184 genes, and upregulation of skin barrier‐related pathways (Figure S3E–G). These results demonstrate that *Yang08* attenuates both systemic and local inflammatory responses in AD.

**FIGURE 6:**
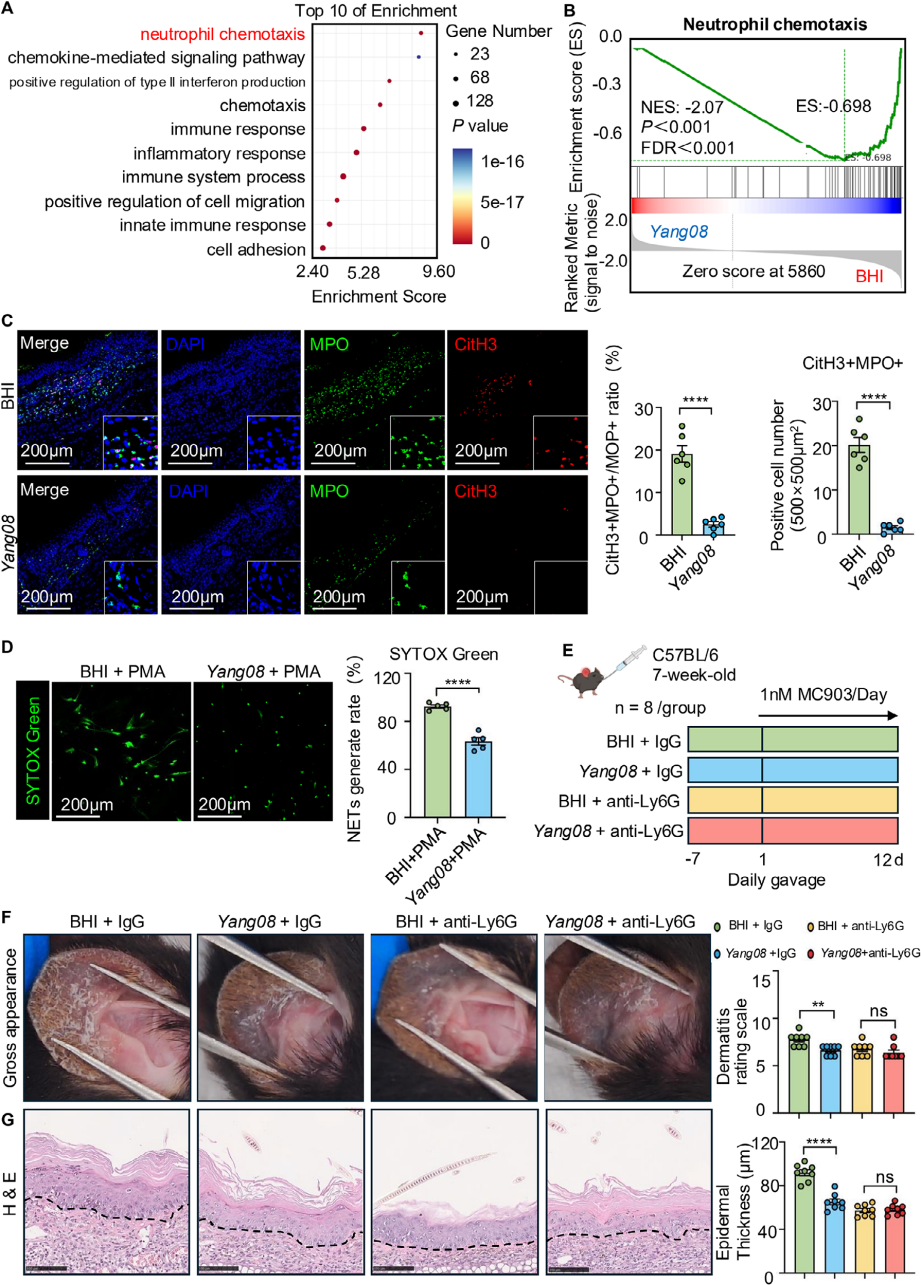
The therapeutic efficacy of *Yang08* depends on functional neutrophils and the suppression of NET formation (A) GO analysis of downregulated genes in *Yang08*‐treated lesional skin, highlighting neutrophil chemotaxis as the top suppressed pathway. (B) Gene Set Enrichment Analysis (GSEA) plot confirming significant inhibition of neutrophil chemotaxis gene sets (NES = ‐0.698, FDR<0.001). (C) Representative immunofluorescence images showing CitH3 (red), MPO (green) and DAPI (blue) staining, and quantification of CitH3+MPO+ cell numbers and their percentage among MPO+ cells in ear skin. (*n* = 6 mice per group). Scale bar, 200 µm. ^****^ *P *< 0.0001 for *Yang08* vs. BHI group by unpaired t‐test. (D) Quantification of NETosis from peripheral blood neutrophils stimulated with PMA *ex vivo*, as measured by SYTOX Green fluorescence (*n* = 5 mice per group). Scale bar, 200 µm. ^****^ *P *< 0.0001 for *Yang08* vs. BHI group by unpaired t‐test. (E) Schematic of in vivo neutrophil depletion using anti‐Ly6G monoclonal antibody. (F, G) Disease parameters in neutrophil‐depleted mice (*n* = 8 mice per group). (F) Representative macroscopic images of ears and clinical dermatitis scores on day 12. (G) Representative H&E‐stained sections of ear skin and quantification of epidermal thickness. Scale bar, 100 µm. For (F, G), ^**^ *P *< 0.01, ^****^ *P *< 0.0001 for *Yang08* + IgG vs. BHI + IgG; ns, not significant for *Yang08* + anti‐Ly6G vs. BHI + anti‐Ly6G by unpaired t‐test. All data are presented as mean ± SEM.

To elucidate the immunoregulatory mechanisms of *Yang08*, unbiased pathway enrichment analysis was conducted, revealing suppression of neutrophil chemotaxis as the top‐ranking biological process (Figure 6A). This was further supported by GSEA, which showed significant inhibition of neutrophil chemotaxis pathways (NES = −0.698, FDR* < *0.001; Figure 6B). Consistent with emerging evidence implicating neutrophil and NETs in amplifying Th2 inflammation in AD [17, 18, 19], we next quantified NETotic activity. Immunofluorescence staining for MPO and CitH3 revealed that *Yang08* decreased not only the percentage of CitH3+MPO+ cells relative to all MPO+ cells but also the absolute count of CitH3+MPO+ cells per visual field in lesional skin (*P < *0.0001; Figure 6C). Subsequently, we isolated peripheral blood neutrophils from both BHI‐ and *Yang08*‐treated mice and stimulated them with PMA. Neutrophils from *Yang08*‐treated mice exhibited a significantly reduced capacity to form NETs compared to those from the BHI‐treated group (*P < *0.0001; Figure 6D). Importantly, the co‐administration of *Yang08* plus *A. muciniphila*, the taxon specifically expanded by *Yang08*, reproduced this NET‐suppressive phenotype in germ‐free mice (*P < *0.0001; Figure S3H).

To determine whether neutrophils are required for *Yang08*’s protection, we depleted them with anti‐Ly6G monoclonal antibody (Figure 6E). Neutrophil depletion abolished the therapeutic differences between the *Yang08* and BHI groups across all evaluated endpoints, including ear thickness, dermatitis scores, epidermal hyperplasia, and mast cell infiltration (all *P *> 0.05; Figure S3I, Figure 6F–G, Figure S3J). Accordingly, immunofluorescence staining for CitH3 showed that neutrophil clearance eliminated CitH3‐positive signals in skin lesions (Figure S3K), confirming that the alleviation of dermatitis by *Yang08* depends on functional neutrophils and the generation of NETs.

### 2.7 NETs Inhibition is Required for *Yang08*‐Mediated Therapeutic Efficacy

**FIGURE 7:**
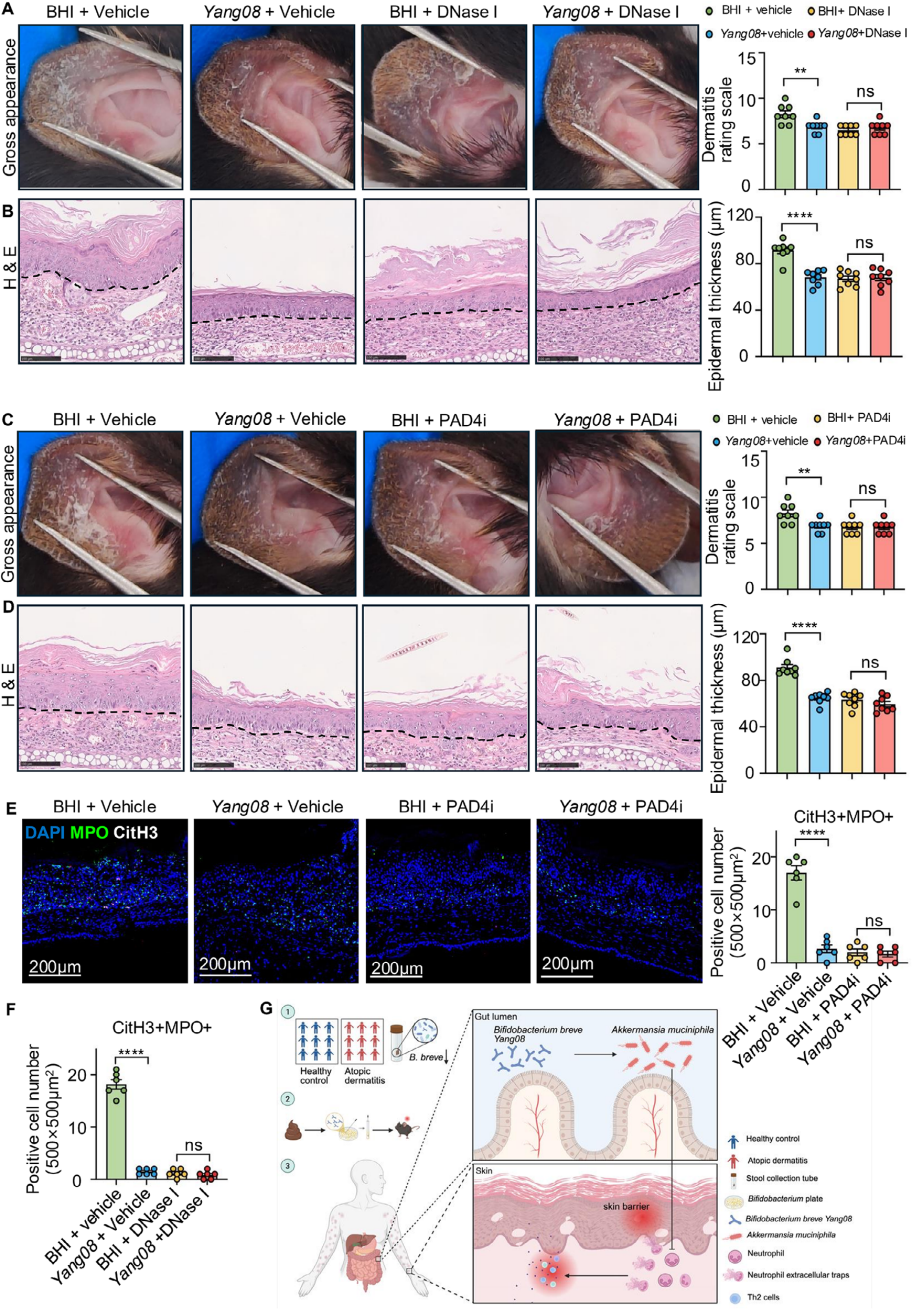
NETs inhibition is required for *Yang08*‐mediated therapeutic efficacy (A, B) Effects of DNase I‐mediated NET degradation on *Yang08*'s protection (*n* = 8 mice per group). (A) Representative macroscopic images of ears and clinical dermatitis scores on day 12. (B) Representative H&E‐stained sections of ear skin and quantification of epidermal thickness. Scale bar, 100 µm. For (A, B), ^**^ *P *< 0.01, ^****^ *P *< 0.0001 for *Yang08* + vehicle vs. BHI + vehicle; ns, not significant for *Yang08* + DNase I vs. BHI + DNase I by unpaired *t*‐test. (C, D) Effects of PAD4 inhibitor (GSK484)‐mediated NETosis inhibition on *Yang08*'s protection (*n* = 8 mice per group). (C) Representative macroscopic images of ears and clinical dermatitis scores on day 12. (D) Representative H&E‐stained sections of ear skin and quantification of epidermal thickness. Scale bar, 100 µm. (E) Representative immunofluorescence images showing CitH3 (red), MPO (green) and DAPI (blue) staining, and quantification of CitH3+MPO+ cells in ear skin lesions from mice treated with or without PAD4 inhibitor. Scale bars, 200 µm. For C‐E, ^**^ *P *< 0.01, ^****^ *P *< 0.0001 for *Yang08* + vehicle vs. BHI + vehicle; ns, not significant for *Yang08* + PAD4i vs. BHI + PAD4i by unpaired t‐test. (F) Quantification of CitH3+MPO+ cells in ear skin lesions from mice with or without DNase I treatment. ^****^ *P *< 0.0001 for *Yang08* + vehicle vs. BHI + vehicle; ns, not significant for *Yang08* + DNase I vs. BHI + DNase I by unpaired *t*‐test. (G) Proposed working model: *Yang08*, depleted in AD patients, alleviates dermatitis by reshaping the gut microbiota (enriching *A. muciniphila*) and inhibiting neutrophil infiltration and NETosis, ultimately restoring skin barrier and reducing inflammation. All data are presented as mean ± SEM.

We next asked whether NETs themselves mediate the benefit. DNase I was administered to the mice to degrade existing NETs (Figure S4A). This enzymatic clearance of NETs effectively abolished the therapeutic advantage of *Yang08* over the BHI control, eliminating the significant differences between the two groups in ear thickness, dermatitis scores, epidermal hyperplasia, and mast cell infiltration (all *P *> 0.05; Figure S4B, C and Figure 7A, B). Similarly, pharmacologic blockade of PAD4‐driven NET formation with GSK484 (Figure S4D) abrogated the *Yang08‐*induced improvements, rendering it indistinguishable from the BHI group across all the disease parameters (all *P *> 0.05; Figure S4E, F and Figure 7C, D). CitH3 immunofluorescence confirmed the efficient elimination of NETs in skin lesions by both interventions (Figure 7E‐F and Figure S44G). Collectively, these loss‐of‐function experiments establish that the degradation or prevention of NETs formation abrogates the therapeutic benefit of *Yang08*. NETs are an essential downstream effector in its mechanism of action.

Finally, a schematic summarizing these mechanisms is presented in Figure 7G: *B. breve* is depleted in AD patients. *B. breve Yang08*, a novel live biotherapeutic, orchestrates gut‐skin axis communication through *A. muciniphila*‐mediated microbiota remodeling and a previously unrecognized blockade of NETs formation, offering new mechanistic insights into AD treatment.

## 3 Discussion

Understanding the interplay between gut microbiota and skin inflammation is essential for advancing therapeutic approaches for AD. In the present study, we establish a causal link between specific gut commensals and the regulation of cutaneous innate immunity. We demonstrate that a novel human isolate, *B. breve Yang08*, alleviates AD not through direct action, but by orchestrating a gut ecosystem shift that enriches *A. muciniphila*. Crucially, we identify the suppression of NETs as the essential downstream effector mechanism of this gut‐skin crosstalk. This finding has profoundly demonstrated the necessity of microbial‐host interactions and underscores critical considerations for the development of next‐generation probiotic therapies in AD [27, 28].

Our initial observation was that *B. breve* is depleted in AD patients. Previous studies have shown that *B. breve* can directly modulate host immunity, such as through indole‐3‐lactic acid mediated suppression of colitis‐associated tumorigenesis [29]. However, a key finding of our work is the strain‐specific and viability‐dependent nature of *Yang08*’s efficacy. Unlike heat‐killed bacteria or cell‐free supernatants, only live *Yang08* conferred protection, suggesting that active colonization is required. More importantly, using antibiotic‐treated and GF mouse models, *Yang08* monotherapy failed but restored protection when co‐administered with *A. muciniphila*, a mucin‐degrading commensal known to enhance gut barrier integrity and suppress systemic inflammation [30, 31]. This suggests a cross‐feeding or synergistic relationship between *B. breve* and *A. muciniphila*. This finding highlights the complexity of microbiome therapeutics.

The most significant conceptual advance of this study is the identification of NETs as the target of gut microbiota modulation in AD. Emerging evidence links gut dysbiosis to enhanced formation of NETs, which in turn amplify type‐2 immune responses and impair epidermal barrier gene expression across multiple inflammatory diseases [32, 33, 34]. We found that *Yang08* treatment significantly reduced the infiltration of NETs formation in skin lesions. Strikingly, the therapeutic benefit of *Yang08* was completely abrogated by neutrophil depletion (anti‐Ly6G), enzymatic NET degradation (DNase I), or pharmacological NET inhibition (PAD4 inhibitor). These “loss‐of‐function” experiments definitively place NET inhibition downstream of the *Yang08*‐*A. muciniphila* axis. *Yang08*‐mediated inhibition of NETs formation represents a previously unrecognized immunomodulatory mechanism for *bifidobacteria*, by disrupting this pathological amplification loop where NETs potentiate Th2 inflammation and skin barrier damage in AD [33, 35]. *A. muciniphila* has been shown to mitigate neutrophil infiltration in other models of acute pancreatitis and alcoholic liver disease [30, 31, 36]. To our knowledge, this is the first study to demonstrate that manipulating the gut microbiota can specifically resolve cutaneous inflammation by preventing NETosis.

The requirement for live *Yang08* and its strict dependence on microbial ecosystem interactions underscores the limitations of current probiotic formulations and establishes criteria for next‐generation live biotherapeutic products. Future study should focus on elucidating the mechanism by which the *Yang08*‐enriched *A. muciniphila* population communicates with skin neutrophils, as well as optimizing dosing strategies based on host microbiome competition. Patients characterized by abnormal neutrophilic activity or *B. breve* depletion may derive particular benefit.

In conclusion, we have characterized a novel “Gut‐*A. muciniphila*‐NET” axis in the pathogenesis and treatment of AD. By validating *B. breve Yang0*8 as a novel isolated strain capable of remodeling the gut microbiota to suppress cutaneous NETosis, this study not only elucidates a fundamental mechanism of gut‐skin communication but also provides a solid scientific foundation for the development of precision‐engineered live biotherapeutics for inflammatory skin diseases such as AD.

## 4 Methods

### 4.1 Human Cohort and Sample Processing

Fecal specimens were collected from 20 patients with moderate to severe AD, diagnosed according to the Hanifin and Rajka criteria (Eczema Area and Severity Index score  > 7), and from 20 age‐ and gender‐ matched healthy controls. Exclusion criteria included the use of systemic immunosuppressants, biologics, antibiotics, or probiotics, as well as any interventions known to modulate the gut microbiota within the preceding month. Samples were immediately flash‐frozen in liquid nitrogen and stored at −80°C until lyophilization and subsequent DNA extraction. The study protocol was approved by the Institutional Review Board of the Dermatology Hospital of Southern Medical University (Approval No. KY2025007), and written informed consent was obtained from all participants prior to sample collection.

### 4.2 Bacterial Isolation and Culture Conditions

A novel *B. breve* strain was isolated from the feces of a healthy donor using selective agar, with purified colonies identified via Sanger sequencing. *B. breve* ATCC 15700, *B. breve Yang08, A. muciniphila* ATCC BAA‐835, and *Escherichia coli* MG1655 (*E. coli*) were cultured anaerobically in brain heart infusion (BHI) broth supplemented with 0.5% α‐lactose monohydrate at 37°C. Live *E. coli* MG1655 was selected as a non‐beneficial commensal control to differentiate the strain‐specific effects of *Yang08* from the general effects of introducing live bacterial biomass into the gut [23, 24, 37, 38]. Bacterial culture medium was collected by centrifugation at 4,000 rpm for 15 min and filtered through a 0.22 µm sterile membrane. Heat‐inactivated *Yang08* was prepared by incubation at 95°C for 20 min, with complete inactivation confirmed by anaerobic plating.

### 4.3 Murine AD Modeling and Intervention

All animal procedures were approved by the South China Agricultural University Animal Ethics Committee (Approval No. 2024F818). Female C57BL/6 mice (6–8 weeks old; Ruige Biological Technology) were housed under specific pathogen‐free (SPF) conditions (22 ± 1°C, 55 ± 10% humidity, 12‐h light/dark cycle) with ad libitum access to sterile water and standard chow. Following a 7‐day acclimatization period, mice were randomly assigned to experimental groups. An AD‐like model was induced by daily topical application of 10 µL of 1 nM calcipotriol (MC903; Sigma–Aldrich) in ethanol to the inner ear surface for 11 consecutive days under brief isoflurane anesthesia. Control animals received an ethanol vehicle alone [39].

Therapeutic interventions were administered via daily oral gavage (200 µL), commencing 7 days prior to MC903 application and continuing throughout the induction period. Treatment groups received either live *Yang08* (2 × 10^8^ CFU), *Yang08* culture supernatant, heat‐inactivated *Yang08* (2 × 10^8^ CFU), *E. coli* MG1655(2 × 10^8^ CFU), or sterile BHI broth (vehicle control). Clinical dermatitis severity‐encompassing erythema, edema, excoriation, and scaling‐was scored at day 12 using a standardized scale (0 = absent, 1 = mild, 2 = moderate, 3 = severe) [40]. Ear thickness was measured using a digital micrometer (Mitutoyo) and spontaneous scratching behavior was quantified from 30‐min video recordings.

For gut microbiota depletion, mice received a broad‐spectrum antibiotic cocktail (ABX) in autoclaved drinking water (containing 0.2 g·L^−^^1^ ampicillin, 0.2 g·L^−^^1^ neomycin, 0.2 g·L^−^^1^ metronidazole, and 0.1 g·L^−^^1^ vancomycin) [25, 26]. The solution was stored at 4°C protected from light. Mice were administered ABX or vehicle control ad libitum for 14 days prior to the initiation of experimental procedures. Subsequently, the mice were administered BHI or *Yang08* via oral gavage in the absence of ABX intervention.

### 4.4 Fecal Microbiota Transplantation (FMT)

FMT was performed as previously described [41]. Recipient mice were pre‐gavaged with the quadruple antibiotic cocktail (ampicillin 200 mg·kg^−1^, neomycin sulfate 200 mg·kg^−1^, metronidazole 200 mg·kg^−1^, and vancomycin 100 mg·kg^−1^) for five consecutive days prior to transplantation. Fresh fecal samples were collected from donor mice treated with either BHI broth or *Yang08*. Fecal pellets were pooled within each donor group, resuspended in sterile PBS (0.125 g·mL^−1^), and administered to recipient mice by oral gavage once daily for five consecutive days. AD induction was initiated following the completion of FMT.

### 4.5 Gnotobiotic Mouse Models

In the ABX‐treated mouse model, mice were pre‐gavaged with a quadruple antibiotic cocktail (as described above) for five consecutive days and subsequently randomly assigned to receive BHI broth, *Yang08*, or *Yang08* combined with *A. muciniphila* during AD induction. Germ‐free (GF) mice were obtained from Shenzhen Gnotobio Biotechnology and randomized into the same three treatment groups. During the AD modeling period, GF mice were gavaged with the indicated treatments once every three days.

### 4.6 Neutrophil and NETs Manipulation

In each experiment, mice were randomly divided into four groups receiving BHI broth or *Yang08* in combination with either vehicle or the indicated intervention. For neutrophil depletion, mice were injected intraperitoneally with an anti‐Ly6G monoclonal antibody (clone 1A8; BioLegend, 127650), with an initial dose of 100 µg on day 1 followed by 50 µg every other day; AD induction commenced on day 2. For DNase I–mediated degradation of extracellular DNA, mice received daily intraperitoneal injections of DNase I (50 mg·kg^−1^; Roche, 10104159001), starting one day prior to AD induction and continuing throughout the modeling period. For inhibition of PAD4 activity, mice were treated with the PAD4 inhibitor GSK484 (4 mg·kg^−1^; MCE, HY‐100514) by daily intraperitoneal injection commencing one day before AD induction and maintained throughout the modeling period. Corresponding vehicle controls and isotype‐matched antibodies were administered to control groups.

### 4.7 Whole‐Genome Sequencing and Analysis

Whole‐genome sequencing was performed by Novogene. Phylogenomic analysis was conducted using the Comprehensive Genome Analysis pipeline of the Bacterial and Viral Bioinformatics Resource Center (BV‐BRC), which constructs phylogenetic trees based on shared single‐copy protein families. A maximum‐likelihood tree was inferred using RAxML with bootstrap support generated within the pipeline.

### 4.8 Immunohistochemistry and Histology

Murine ear specimens were fixed in 10% neutral‐buffered formalin for 24 h at room temperature, followed by paraffin embedding and sectioning at 5µm thickness. Sections were dewaxed in xylene, rehydrated, and stained with hematoxylin and eosin (H&E) for general histopathological evaluation. Mast cells were stained with toluidine blue and counted in five high‐power fields per section. For immunofluorescence staining, heat‐induced epitope retrieval was performed using microwave decloaking in citrate buffer (pH 9.0). After blocking with 5% bovine serum albumin for 1 h at room temperature, sections were incubated overnight at 4°C with primary antibodies against myeloperoxidase (MPO; R&D, AF3667; 1:800) and citrullinated histone H3 (CitH3) (Abcam, ab5103; 1:1000). Sections were subsequently incubated with Alexa Fluor 647‐conjugated donkey anti‐goat IgG secondary antibody (Abcam, ab150131; 1:1000) and Alexa Fluor 555‐conjugated donkey anti‐rabbit IgG secondary antibody (Abcam, ab15074; 1:1000) for 1 h followed by DAPI nuclear counterstaining. Images were acquired using a Nikon confocal microscope.

### 4.9 Fecal Metagenomic Sequencing

Fresh fecal pellets were collected from mice, immediately flash‐frozen in liquid nitrogen, and stored at −80°C. Genomic DNA extraction was performed by Novogene. Sequencing libraries were prepared and run on the Illumina platform. Raw reads were preprocessed by fastp, and host‐derived sequences were removed to generate clean data. Metagenome assembly was performed with MEGAHIT, followed by gene prediction using MetaGeneMark. Taxonomic annotation was conducted using Kraken2 against the standard database.

### 4.10 Bulk RNA Sequencing and Analysis

Total RNA was extracted from lesional skin tissues using TRIzol reagent (Thermo Fisher Scientific). Libraries were subjected to paired‐end sequencing (150 bp reads) on the Illumina NovaSeq PE150 platform. Raw reads were quality‐filtered using fastp software and aligned to the reference genome using Hisat2. Differential expression analysis was conducted with DESeq2 using a *P* value threshold of 0.05. Gene set enrichment analysis was performed using the ClusterProfiler R package.

### 4.11 Isolation and Stimulation of Mice Neutrophils

EDTA‐anticoagulated peripheral blood was collected from mice treated with either BHI broth or *Yang08*. Neutrophils were isolated using a commercial neutrophil isolation kit (Solarbio, Cat. No. 9201) according to the manufacturer's instructions, resuspended in complete medium, and seeded into 48‐well plates. Neutrophils derived from BHI broth‐ or *Yang08*‐treated mice were stimulated with phorbol 12‐myristate 13‐acetate (PMA 250 nM, Sigma–Aldrich, Cat. No. P1585) for 4 h at 37 °C. After stimulation, cells were stained with SYTOX Green nucleic acid dye (1.25 µm, Invitrogen, Cat. No. S7020) for NETs visualization and imaged using a Nikon confocal microscope.

### 4.12 Statistical Analysis

Continuous variables were presented as mean ± SEM. Comparisons between two groups were performed using unpaired Student's *t* ‐ tests, while comparisons among multiple groups were conducted using one‐way ANOVA followed by Dunnett's multiple comparisons test. All statistical analyses were performed using GraphPad Prism 10.0, with *P < *0.05 considered statistically significant.

## Funding

This study was supported by the National Natural Science Foundation of China grants 32470958 (QL), 82403246(QL) and 82574001 (ZFL); Guangdong Provincial Science and Technology Plan Project (2025A04J4030)

## Ethics Approval and Consent to Participate

Human participants provided written informed consent prior to sample collection, following protocols reviewed by the Dermatology Hospital of Southern Medical University (Approval No. KY2025007). Animal experiments were approved by the Institutional Animal Care and Use Committee of South China Agricultural University Animal Ethics Committee (Approval No. 2024F818).

## Conflicts of Interest

The authors declare no conflicts of interest

## Consent

All participants consented to publication of research findings.

## Supporting information




**Supporting File**: advs74130‐sup‐0001‐SuppMat.pdf.

## Data Availability

The datasets generated and analyzed in this study are available from the corresponding author upon reasonable request.

## References

[1] S. M. Langan , A. D. Irvine , and S. Weidinger , “Atopic Dermatitis,” The Lancet 396 (2020): 345–360. 10.1016/S0140-6736(20)31286-1 32738956

[2] S. Ständer , “Atopic Dermatitis,” New England Journal of Medicine 384 (2021): 1136–1143. 33761208 10.1056/NEJMra2023911

[3] S. Meledathu , M. P. Naidu , and P. M. Brunner , “Update on Atopic Dermatitis,” Journal of Allergy and Clinical Immunology 155 (2025): 1124–1132. 39855361 10.1016/j.jaci.2025.01.013

[4] M. Schmuth , S. Eckmann , V. Moosbrugger‐Martinz , et al., “Skin Barrier in Atopic Dermatitis,” Journal of Investigative Dermatology 144 (2024): 989–1000. 38643989 10.1016/j.jid.2024.03.006

[5] E. Guttman‐Yassky , Y. Renert‐Yuval , and P. M. Brunner , “Atopic Dermatitis,” The Lancet 405 (2025): 583–596. 10.1016/S0140-6736(24)02519-4 39955121

[6] H. Li , Z. Zhang , H. Zhang , Y. Guo , and Z. Yao , “Update on the Pathogenesis and Therapy of Atopic Dermatitis,” Clinical Reviews in Allergy & Immunology 61 (2021): 324–338. 34338977 10.1007/s12016-021-08880-3

[7] M. J. Alam , L. Xie , Y.‐A. Yap , F. Z. Marques , and R. Robert , “Manipulating Microbiota to Treat Atopic Dermatitis: Functions and Therapies,” Pathogens 11 (2022): 642. 35745496 10.3390/pathogens11060642 PMC9228373

[8] S. Mirali and A. M. Drucker , “New Topical and Systemic Treatments for Atopic Dermatitis,” Clinical & Experimental Allergy 55 (2025): 1057–1069. 40836559 10.1111/cea.70136 PMC12617513

[9] X. Lin , Z. Yu , Y. Liu , et al., Imeta 4 (2025): 270.

[10] X. Liu , Y. Luo , X. Chen , et al., “Fecal Microbiota Transplantation against Moderate‐to‐Severe Atopic Dermatitis: a Randomized, Double‐Blind Controlled Explorer Trial,” Allergy 80 (2025): 1377–1388. 39470619 10.1111/all.16372

[11] M. Lima and L. C. Paulino , “Oral Postbiotics as a Therapeutic Strategy for Atopic Dermatitis: a Systematic Review of Randomized Controlled Trials,” Journal of the american nutrition association 43 (2024): 139–146. 37459239 10.1080/27697061.2023.2232021

[12] L. Carucci , R. Nocerino , L. Paparo , et al., “Therapeutic Effects Elicited by the Probiotic Lacticaseibacillusrhamnosus gg in Children with Atopic Dermatitis. The Results of the ProPAD Trial,” Pediatric Allergy and Immunology 33 (2022): 13836. 10.1111/pai.13836 PMC9542056 36003050

[13] L. Yang , D. Li , S. Sun , et al., “Dupilumab Therapy Improves Gut Microbiome Dysbiosis and Tryptophan Metabolism in Chinese Patients with Atopic Dermatitis,” International Immunopharmacology 131 (2024): 111867. 38493690 10.1016/j.intimp.2024.111867

[14] C.‐C. Chiang , W.‐J. Cheng , J. R. M. S. Dela Cruz , et al., “Neutrophils in Atopic Dermatitis,” Clinical Reviews in Allergy & Immunology 67 (2024): 21–39. 39294505 10.1007/s12016-024-09004-3 PMC11638293

[15] D. F. Choy , D. K. Hsu , D. Seshasayee , et al., “Comparative Transcriptomic Analyses of Atopic Dermatitis and Psoriasis Reveal Shared Neutrophilic Inflammation,” Journal of Allergy and Clinical Immunology 130 (2012): 1335–1343. 22920495 10.1016/j.jaci.2012.06.044 PMC3511596

[16] D. Pavlenko , Z. T. Seven , L. Bystrom , et al., “Crisaborole Inhibits Itch and Pain by Preventing Neutrophil Infiltration in a Mouse Model of Atopic Dermatitis,” Acta Dermato‐Venereologica 103 (2023): adv13382. 37605895 10.2340/actadv.v103.13382 PMC10461178

[17] C. M. Walsh , R. Z. Hill , J. Schwendinger‐Schreck , et al., “Neutrophils Promote CXCR3‐dependent Itch in the Development of Atopic Dermatitis,” eLife 8 (2019): 48448. 10.7554/eLife.48448 PMC6884397 31631836

[18] C. Zheng , T. Cao , C. Ye , and Y. Zou , “Neutrophil Recruitment by CD4 Tissue‐resident Memory T Cells Induces Chronic Recurrent Inflammation in Atopic Dermatitis,” Clinical Immunology 256 (2023): 109805. 37832861 10.1016/j.clim.2023.109805

[19] T. Liao , C. Lin , Y. Chen , and K. Tang , “Reduced miR ‐223 Increases Blood Neutrophil Extracellular Trap and Promotes Skin Inflammation in Atopic Dermatitis,” Allergy 78 (2023): 3252–3254. 37365911 10.1111/all.15794

[20] W. Fong , Q. Li , F. Ji , et al., “Lactobacillus Gallinarum ‐derived Metabolites Boost Anti‐PD1 Efficacy in Colorectal Cancer by Inhibiting Regulatory T Cells through Modulating IDO1/Kyn/AHR Axis,” Gut 72 (2023): 2272. 37770127 10.1136/gutjnl-2023-329543 PMC10715476

[21] H. C.‐H. Lau , X. Zhang , F. Ji , et al. eBioMedicine (2024): 100. 10.1016/j.ebiom.2023.104952 PMC10801313 38176203

[22] H. Tsoi , E. S. H. Chu , X. Zhang , et al., “Peptostreptococcus Anaerobius Induces Intracellular Cholesterol Biosynthesis in Colon Cells to Induce Proliferation and Causes Dysplasia in Mice,” Gastroenterology 152 (2017): 1419–1433. 28126350 10.1053/j.gastro.2017.01.009

[23] Q. Li , W. Hu , W. X. Liu , et al., “Streptococcus Thermophilus Inhibits Colorectal Tumorigenesis through Secreting β‐Galactosidase,” Gastroenterology 160 (2021): 1179–1193. 32920015 10.1053/j.gastro.2020.09.003

[24] Q. Li , H. Chan , W.‐X. Liu , et al., “Carnobacterium Maltaromaticum Boosts Intestinal Vitamin D Production to Suppress Colorectal Cancer in Female Mice,” Cancer Cell 41 (2023): 1450–1465. 37478851 10.1016/j.ccell.2023.06.011

[25] H. Gou , H. Su , D. Liu , et al., “Traditional Medicine Pien Tze Huang Suppresses Colorectal Tumorigenesis through Restoring Gut Microbiota and Metabolites,” Gastroenterology 165 (2023): 1404–1419. 37704113 10.1053/j.gastro.2023.08.052

[26] J. Yang , H. Wei , Y. Zhou , et al., “High‐Fat Diet Promotes Colorectal Tumorigenesis through Modulating Gut Microbiota and Metabolites,” Gastroenterology 162 (2022): 135–149. 34461052 10.1053/j.gastro.2021.08.041

[27] X. Chen , Y. Chen , C. Stanton , et al., “Dose–Response Efficacy and Mechanisms of Orally Administered Bifidobacterium Breve CCFM683 on IMQ‐Induced Psoriasis in Mice,” Nutrients 15 (2023): 1952. 37111171 10.3390/nu15081952 PMC10143451

[28] Z. Fang , T. Pan , L. Li , et al., “Bifidobacterium Longum Mediated Tryptophan Metabolism to Improve Atopic Dermatitis via the Gut‐skin Axis,” Gut Microbes 14 (2022): 2044723. 35239463 10.1080/19490976.2022.2044723 PMC8903757

[29] Y. Li , Q. Li , R. Yuan , Y. Wang , C. Guo , and L. Wang , “Bifidobacterium Breve ‐derived Indole‐3‐lactic Acid Ameliorates Colitis‐associated Tumorigenesis by Directing the Differentiation of Immature Colonic Macrophages,” Theranostics 14 (2024): 2719–2735. 38773969 10.7150/thno.92350 PMC11103503

[30] L.‐J. Wang , Y.‐L. Jin , W.‐L. Pei , et al., “Amuc_1100 pretreatment Alleviates Acute Pancreatitis in a Mouse Model through Regulating Gut Microbiota and Inhibiting Inflammatory Infiltration,” Acta Pharmacologica Sinica 45 (2024): 570–580. 38012292 10.1038/s41401-023-01186-4 PMC10834448

[31] C. Grander , T. E. Adolph , V. Wieser , et al., “Recovery of Ethanol‐induced Akkermansia Muciniphila Depletion Ameliorates Alcoholic Liver Disease,” Gut 67 (2018): 891. 28550049 10.1136/gutjnl-2016-313432

[32] D. A. Waizman , S. Ghosh , and C. V. Rothlin , “Bringing on the Itch,” eLife 8 (2019): 52931. 10.7554/eLife.52931 PMC6884404 31782733

[33] X. Tong , S. H. Kim , L. Che , J. Park , J. Lee , and T.‐G. Kim , “Foxp3+ Treg Control Allergic Skin Inflammation by Restricting IFN‐γ‐driven Neutrophilic Infiltration and NETosis,” Journal of Dermatological Science 115 (2024): 2–12. 38845244 10.1016/j.jdermsci.2024.05.002

[34] M. Toussaint , D. J. Jackson , D. Swieboda , et al., “Host DNA Released by NETosis Promotes Rhinovirus‐induced Type‐2 Allergic Asthma Exacerbation,” Nature Medicine 23 (2017): 681–691. 10.1038/nm.4332 PMC5821220 28459437

[35] J. Focken , J. Scheurer , A. Jäger , et al., “Neutrophil Extracellular Traps Enhance S. aureus Skin Colonization by Oxidative Stress Induction and Downregulation of Epidermal Barrier Genes,” Cell Reports 42 (2023): 113148. 37733587 10.1016/j.celrep.2023.113148

[36] Z. Tian , Y. Zhang , Z. Zheng , et al., “Gut Microbiome Dysbiosis Contributes to Abdominal Aortic Aneurysm by Promoting Neutrophil Extracellular Trap Formation,” Cell Host & Microbe 30 (2022): 1450–1463. 36228585 10.1016/j.chom.2022.09.004

[37] A. C. Y. Su , X. Ding , H. C. H. Lau , et al., “Lactococcus Lactis HkyuLL 10 Suppresses Colorectal Tumourigenesis and Restores Gut Microbiota through Its Generated Alpha‐mannosidase,” Gut 73 (2024): 1478. 38599786 10.1136/gutjnl-2023-330835 PMC11347254

[38] N. Sugimura , Q. Li , E. S. H. Chu , et al., “Lactobacillus Gallinarum Modulates the Gut Microbiota and Produces Anti‐cancer Metabolites to Protect against Colorectal Tumourigenesis,” Gut 71 (2022): 2011–2021. 10.1136/gutjnl-2020-323951 PMC9484392 34937766

[39] M. J. Alam , L. Xie , Y. A. Yap , and R. Robert , “A Mouse Model of MC903‐Induced Atopic Dermatitis,” Current Protocols 3 (2023): 695. 10.1002/cpz1.695 36913546

[40] J. Han , X. Cai , S. Qin , et al., “TMEM232 promotes the Inflammatory Response in Atopic Dermatitis via the Nuclear Factor‐κB and Signal Transducer and Activator of Transcription 3 Signalling Pathways,” British Journal of Dermatology 189 (2023): 195–209. 36928730 10.1093/bjd/ljad078

[41] D. Li , Y. Chen , M. Wan , et al., “Oral Magnesium Prevents Acetaminophen‐induced Acute Liver Injury by Modulating Microbial Metabolism,” Cell Host & Microbe 32 (2024): 48–62. 38056458 10.1016/j.chom.2023.11.006

