# Supplementary material for: Bifidobacterium Breve Yang08 Alleviates Atopic Dermatitis By Enriching Akkermansia Muciniphila and Inhibiting Neutrophil Extracellular Traps Formation In Mice
Source: Adv Sci (Weinh). 2026 Feb 8;13(20):e18588. doi: 10.1002/advs.202518588 (PMC13067854; doi:10.1002/advs.202518588)
Supplement: Supplementary file 1 — Supporting File: advs74130‐sup‐0001‐SuppMat.pdf. [file ADVS-13-e18588-s001.pdf]

Supplementary Method

Enzyme-linked immunosorbent assay (ELISA)

A mouse-specific uncoated IgE ELISA kit (Invitrogen, 88-50460) was used with protocol modifications. Plasma samples were diluted 25× in the provided buffer. Microplates were coated with capture antibody overnight at 4°C and subsequently blocked. 50 μL of standards (2-fold serial dilution) and diluted samples were added in duplicate. Following 2-hour incubation with continuous agitation, plates were washed and sequentially incubated with biotinylated antibody (60 min) and streptavidin-HRP (30 min). TMB substrate reaction (15 min) was stopped by adding stop solution. Optical density readings at 450 nm and concentrations calculated using a 4-parameter logistic regression curve. All samples per experiment were assayed on the same plate.

Flow Cytometric Immunophenotyping

Single-cell suspensions were obtained from mouse ears by mechanical dissociation followed by enzymatic digestion in RPMI-1640 medium supplemented with collagenase IV (2 mg/mL; Yeasen, 40510ES60) and DNase I (0.5 mg/mL; Roche, 10104159001) for 1 hour at 37°C. The resulting suspensions were filtered through 70 μm cell strainers to remove debris. For intracellular cytokine detection, cells were stimulated with Cell Stimulation Cocktail (eBioscience, 00-4975-93) for 4 hours at 37°C. Surface staining was performed on ice for 30 minutes in the dark using Fixable Viability Stain 780 (BD, 564997) and fluorochrome-conjugated antibodies against the following surface markers (FITC anti-mouse CD45, BioLegend, 147710; PE anti-mouse CD3, BioLegend, 100206; PerCP/Cyanine5.5 anti-mouse CD4, BioLegend, 100540). Intracellular staining (Brilliant Violet 605 anti-mouse IL-4, BioLegend, 504126) was performed using a fixation and permeabilization kit (BD, 554722) with a 1-hour incubation at room temperature in the dark. Data were acquired using a BD FACS Celesta flow cytometer (BD Biosciences), with subsequent analysis using FlowJo v10.8.1 software.

Supplementary Figures

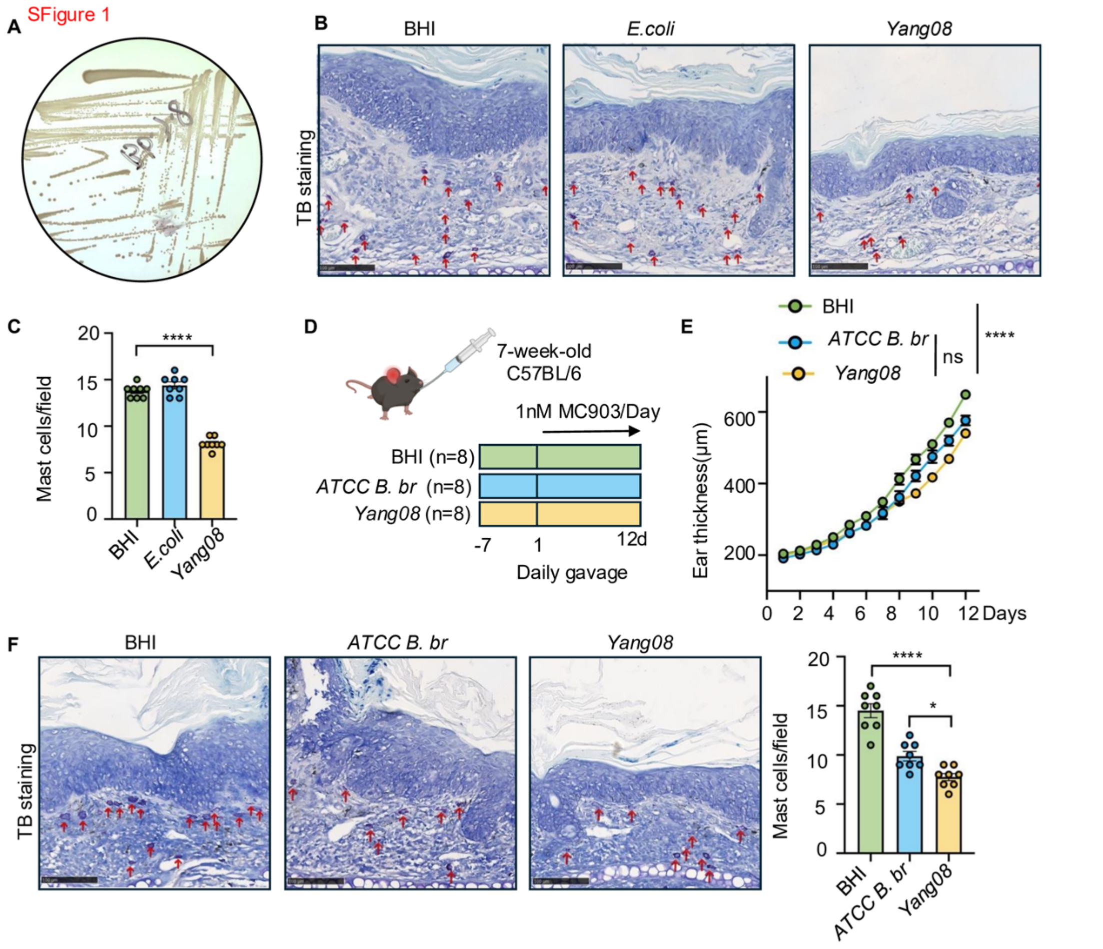

Supplementary Figure 1. Isolation, characterization, and comparative efficacy of *Bifidobacterium breve Yang08*.

(A) Representative colony morphology of the isolated *B. breve Yang08* strain on selective agar under anaerobic conditions.

(B, C) Histopathological assessment of mast cell infiltration. (B) Representative toluidine blue-stained sections of ear skin. Red arrows indicate mast cells. Scale bar, 100 μm. (C) Quantification of mast cell counts per high-power field (HPF) (n=8 mice per group). *****P*<0.0001 for *Yang08* vs. BHI group by one-way ANOVA with Dunnett's test.

(D) Schematic of the experimental design comparing the novel *Yang08* strain with the standard *B. breve* ATCC 15700 strain.

(E, F) Comparative therapeutic efficacy between *B. breve Yang08* and the standard ATCC 15700 strain. (n=8 mice per group) (E) Ear thickness over time in mice treated with BHI, ATCC *B. br*, or *Yang08*. ****P<0.001 for BHI vs. *Yang08*; ns, not significant for ATCC *B. br* vs. *Yang08* by two-way ANOVA. (F) Representative toluidine blue-stained sections of ear skin and quantification of mast cells per HPF. Red arrows indicate mast cells. Scale bar, 100 μm. *P<0.05, ***P<0.0001 vs. *Yang08* group by one-way ANOVA with Dunnett's test.

All data are presented as mean ± SEM.

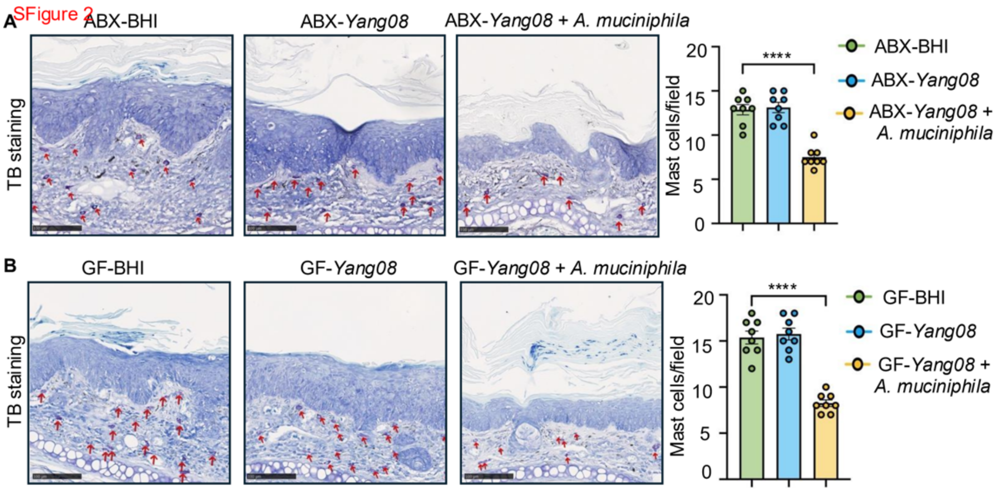

Supplementary Figure 2. ***A. muciniphila*** **is required for** ***Yang08*****'s efficacy in ABX-treated and germ-free mice**

(A) Representative toluidine blue-stained sections of ear skin from antibiotic-treated mice receiving BHI, *Yang08*, or *Yang08* + *A. muciniphila*, and quantification of mast cell counts per HPF (n=8 mice per group). Red arrows indicate mast cells. Scale bar, 100 µm.

(B) Representative toluidine blue-stained sections of ear skin from germ-free mice receiving BHI, *Yang08*, or *Yang08* + *A. muciniphila*, and quantification of mast cell counts per HPF (n=8 mice per group). Red arrows indicate mast cells. Scale bar, 100 µm. For (A-B), ****P<0.0001 for *Yang08* + *A. muciniphila* vs. BHI group by one-way ANOVA with Dunnett's test.

All data are presented as mean ± SEM.

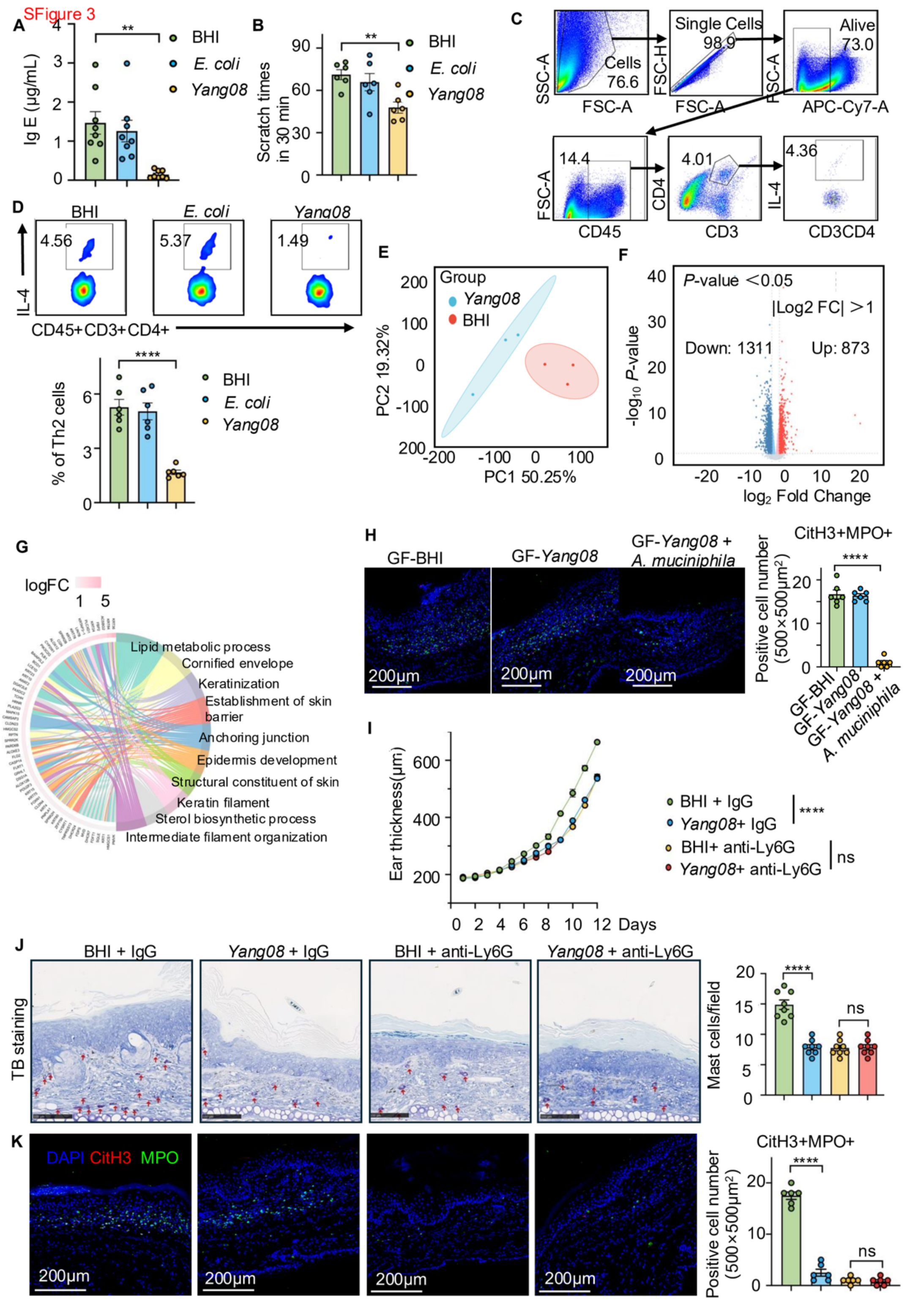

Supplementary Figure 3. The therapeutic efficacy of *Yang08* depends on functional neutrophils and the suppression of NET formation

(A, B) Systemic immune parameters. (A) Plasma total IgE levels measured by ELISA (n=8 mice per group). (B) Number of spontaneous scratching bouts during a 30-

minute observation period (n=6 mice per group). ***P*<0.01 for *Yang08* vs. BHI group by one-way ANOVA with Dunnett's test.

(C, D) Skin Th2 cell infiltration. (C) Representative flow cytometry gating strategy for identifying CD45+CD3+CD4+IL-4+ T cells from ear skin single-cell suspensions. (D) Quantification of IL-4+CD4+ T cell frequency (n=6 mice per group).

*****P*<0.0001 for *Yang08* vs. BHI group by one-way ANOVA with Dunnett's test.

(E-G) Skin transcriptomic analysis. (E) Principal component analysis (PCA) plot of RNA-seq data from skin lesions. (F) Volcano plot showing differentially expressed genes (DEGs) between *Yang08* and BHI groups (log2 fold change > |1|, *P* value < 0.05). Red, upregulated genes; blue, downregulated genes. (G) Reactome pathway enrichment analysis of genes upregulated in *Yang08*-treated skin.

(H) Representative immunofluorescence images showing CitH3 (red), MPO (green) and DAPI (blue) staining, and quantification of CitH3+MPO+ cells in ear skin lesions from germ-free mice. Scale bar, 200 µm. *****P*<0.0001 for *Yang08* + *A. muciniphila* vs. BHI group by one-way ANOVA with Dunnett's test.

(I) Ear thickness over time in mice treated with IgG or anti-Ly6G (n=8 mice per group). *****P*<0.0001 for *Yang08* + IgG vs. BHI + IgG; ns, not significant for *Yang08* + anti-Ly6G vs. BHI + anti-Ly6G by two-way ANOVA.

(J) Representative toluidine blue-stained sections of ear skin from mice treated with IgG or anti-Ly6G, and quantification of mast cell counts per HPF. Red arrows indicate mast cells. Scale bar, 100 µm. (n=8 mice per group).

(K) Representative immunofluorescence images showing CitH3 (red), MPO (green) and DAPI (blue) staining, and quantification of CitH3+MPO+ cells in ear skin lesions from mice treated with anti-Ly6G or IgG. (n=6 mice per group) Scale bar, 200 µm. For (J-K), *****P*<0.0001 for *Yang08* + IgG vs. BHI + IgG; ns, not significant for *Yang08* + anti-Ly6G vs. BHI + anti-Ly6G by unpaired t-test.

All data are presented as mean ± SEM.

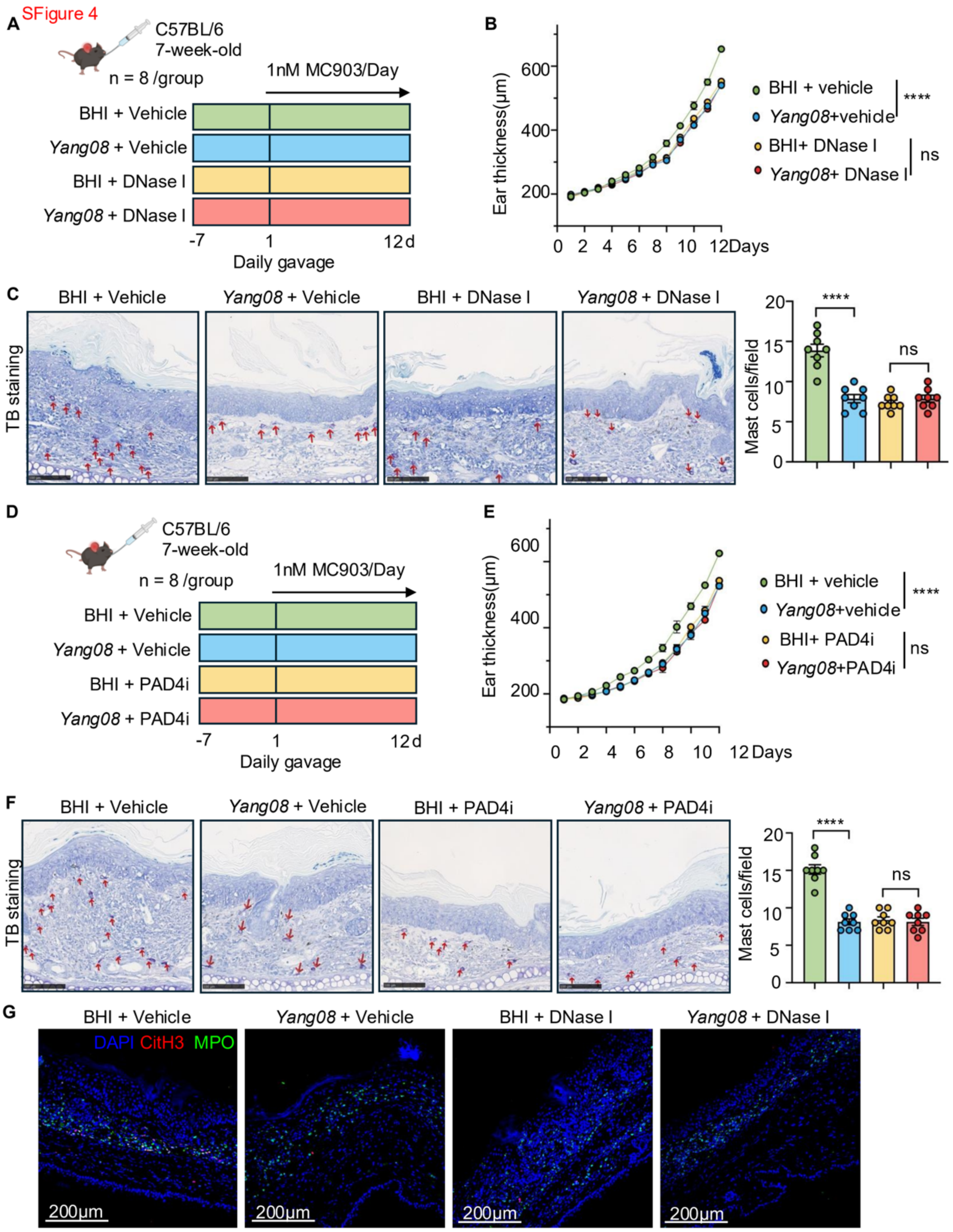

Supplementary Figure 4. NETs inhibition is required for *Yang08*-mediated therapeutic efficacy

(A-C) DNase I intervention. (n=8 mice per group) (A) Schematic of the DNase I treatment protocol. (B) Ear thickness measurement over time in mice treated with DNase I or vehicle. ****P<0.0001 for *Yang08* + vehicle vs. BHI + vehicle; ns, not

significant for *Yang08* + DNase I vs. BHI + DNase I by two-way ANOVA. (C) Representative toluidine blue-stained ear skin sections from mice treated with DNase I or vehicle, and quantification of mast cell counts per HPF. Red arrows indicate mast cells. Scale bar, 100 µm. ****P<0.0001 for *Yang08* + vehicle vs. BHI + vehicle; ns, not significant for *Yang08* + DNase I vs. BHI + DNase I by unpaired t-test.

(D-F) PAD4 inhibitor intervention. (n=8 mice per group) (D) Schematic of the GSK484 treatment protocol. (E) Ear thickness measurement over time in mice treated with GSK484 or vehicle. ****P<0.0001 for *Yang08* + vehicle vs. BHI + vehicle; ns, not significant for *Yang08* + PAD4i vs. BHI + PAD4i by two-way ANOVA. (F) Representative toluidine blue-stained ear skin sections from mice treated with PAD4i or vehicle, and quantification of mast cells per HPF. Red arrows indicate mast cells. Scale bar, 100 µm. ****P<0.0001 for *Yang08* + vehicle vs. BHI + vehicle; ns, not significant for *Yang08* + PAD4i vs. BHI + PAD4i by unpaired t-test.

(G) Representative immunofluorescence images showing CitH3 (red), MPO (green) and DAPI (blue) staining in ear skin lesions from mice treated with DNase I or vehicle. Scale bar, 200 µm.

All data are presented as mean ± SEM.
